# Can language enhance physical therapists’ willingness to follow *Choosing Wisely* recommendations? A best-worst scaling study

**DOI:** 10.1016/j.bjpt.2023.100534

**Published:** 2023-08-14

**Authors:** Priti Kharel, Joshua R. Zadro, Giovanni Ferreira, Martin Howell, Kirsten Howard, Sally Wortley, Charlotte McLennan, Christopher G. Maher

**Affiliations:** a Institute for Musculoskeletal Health, Sydney Local Health District, Sydney, Australia; b School of Public Health, Faculty of Medicine and Health, The University of Sydney, Sydney, Australia

**Keywords:** Choosing wisely, Language, Low-value care, Overuse, Physical therapy

## Abstract

**Background:**

Choosing Wisely recommendations could reduce physical therapists’ use of low-value care.

**Objective:**

To investigate whether language influences physical therapists’ willingness to follow the Australian Physiotherapy Association's (APA) Choosing Wisely recommendations.

**Design:**

Best-worst Scaling survey

**Methods:**

The six original APA Choosing Wisely recommendations were modified based on four language characteristics (level of detail, strength- qualified/unqualified, framing, and alternatives to low-value care) to create 60 recommendations. Physical therapists were randomised to a block of seven choice tasks, which included four recommendations. Participants indicated which recommendation they were most and least willing to follow. A multinomial logistic regression model was used to create normalised (0=least preferred; 10=most preferred) and marginal preference scores.

**Results:**

215 physical therapists (48.5% of 443 who started the survey) completed the survey. Participants' mean age (SD) was 38.7 (10.6) and 47.9% were female. Physical therapists were more willing to follow recommendations with more detail (marginal preference score of 1.1) or that provided alternatives to low-value care (1.3) and less willing to follow recommendations with negative framing (−1.3). The use of qualified (‘don't routinely’) language (vs. unqualified - ‘don't’) did not affect willingness. Physical therapists were more willing to follow recommendations to avoid imaging for non-specific low back pain (3.9) and electrotherapy for low back pain (3.8) vs. recommendation to avoid incentive spirometry after upper abdominal and cardiac surgery.

**Conclusion:**

Physical therapists were more willing to follow recommendations that provided more detail, alternatives to low-value care, and were positively framed. These findings can inform the development of future Choosing Wisely recommendations and could help reduce low-value physical therapy.

## Highlights

• Choosing Wisely recommendations could reduce low-value care in physical therapy.
• Optimizing language could increase implementation of the recommendations.
• Physical therapists were less willing to follow negatively framed recommendations.
• They were most willing to follow recommendations with high detail.
• They were more willing to follow recommendations with alternatives to low-value care.

## Introduction

Choosing Wisely is a global initiative to reduce low-value care;^1^ care that provides little-to-no benefit or whose potential harm exceeds the probable benefit.^2^ Many physical therapists fail to provide evidence-based care when managing patients with musculoskeletal conditions^3^ and this is not improving over time.^4^ There is a need for low-cost strategies to help physical therapists provide recommended care to people with musculoskeletal conditions. Evidence suggests the Choosing Wisely campaign has helped reduce overuse in several areas of medicine.^5^, ^6^, ^7^ Choosing Wisely recommendations are brief statements intended to guide clinicians away from providing low-value care. Over 250 professional societies worldwide (32 societies in Australia) have contributed to over 1300 Choosing Wisely recommendations targeting low-value tests and treatments.^8^ The recommendations vary across countries with some countries having more recommendations than others (e.g. United States, *n* = 535; Australia, *n* = 172). Globally, there are over 120 physical therapy associations, but only four (associations in the United States, Australia, Canada, and Brazil) have joined the campaign and published their ‘do-not-do’ list of tests and treatments. This includes the Australian Physiotherapy Association (APA) (which published six recommendations in 2015) and the Brazilian Association of Traumatology and Orthopaedic Physical therapy (ABRAFITO) (which published five different recommendations in 2020).

There is marked variation in the language of recommendations,^9^ which reflects a lack of guidance and uncertainty on how language could be used to support adoption amongst clinicians. The language of some recommendations is stronger or more qualified than others (e.g., ‘don't do X’ vs. ‘don't routinely do X’). Some recommendations simply discourage low-value care, while others also offer encouragement to adopt high-value care (‘don't do X’ vs. ‘don't do X, instead, do Y’). Evidence suggests clinicians may be more willing to follow Choosing Wisely recommendations if the recommendations were more detailed,^10^, ^11^, ^12^ used unqualified language (‘must’ or ‘don't’)^13^ and provided encouragement to deliver evidence-based care, particularly when discouraging the use of an intervention.^14^ No studies have explored how the language of Choosing Wisely recommendations supports or discourages their adoption amongst clinicians.

A previous qualitative study exploring physical therapists’ opinions on the APA Choosing Wisely recommendations found that the language of the recommendations was one of the barriers to their adoption in practice.^15^ To build on these findings, we wanted to quantitatively investigate whether language influenced physical therapists’ willingness to follow the APA's Choosing Wisely recommendations and understand whether modifying the language of these recommendations had the potential to increase their adoption and reduce low-value care. The aim of our study was to investigate whether language influenced physical therapists’ willingness to follow the APA's Choosing Wisely recommendations and investigate which characteristics of language affect their willingness to follow the recommendations. We hypothesised that recommendations with more detail, unqualified language, positive framing, and alternatives to low-value care would increase physical therapists’ willingness to follow them compared to recommendations with less detail, qualified language, negative framing, and no alternatives, respectively.

## Methods

### Participant selection and recruitment

We recruited practicing physical therapists with no restrictions on age, sex, clinical experience, area of speciality, or country of practice. The APA included a study invitation in two of their monthly newsletters and the Sydney Local Health District sent out study invitations via email to physical therapists working at Concord Hospital and Royal Prince Alfred Hospital. We also posted the study invitation on Facebook and Twitter. The invitation briefly outlined the purpose of the study and included a hyperlink that directed potentially interested physical therapists to complete the survey. Consent was obtained from all participants who completed the survey. Ethics approval was granted by Review Committee (Royal Prince Alfred Hospital Zone) of the Sydney Local Health District (protocol number: X19–0175 & 2019/ETH1151).

### Data collection

The survey (Supplementary Material - File S1) was administered online using Qualtrics, an online survey platform. Participants rated their familiarity with the APA's Choosing Wisely recommendations (extremely familiar, very familiar, moderately familiar, slightly familiar, and not familiar at all). Participants then completed the best-worst-scaling survey (see section 2.4). The demographic data were collected at the end of the survey where participants provided data on their age (categorised as 20–29, 30–39 and 40+), sex, country of practice, years of experience (categorised as 1–4 years, 5–9 years and 10+ years), clinical area of interest (musculoskeletal, cardiorespiratory, neurological, and other), work setting (private practice, public hospital, private hospital, aged care, sports team, and other), involvement in research (Yes/No), teaching and other professional activities (Yes/No). The survey was open from September to December 2019.

### Survey design

The six original APA Choosing Wisely recommendations use largely similar language. No recommendations used positive framing (‘do X’) or provide alternatives to low-value care. All recommendations outline ‘what’ needs to be done, and none outlined ‘why’ the recommendation is important and ‘who’ the recommendation is targeted towards. Although Choosing Wisely recommendations from the APA should target physical therapists, there is evidence that recommendations from some professional associations target members of other associations.^16^ Specifying ‘who’ the recommendation is targeted towards could therefore be valuable. The only difference in language between the recommendations is that some use unqualified language (‘don't do X’) while others use qualified language (‘avoid’, ‘don't routinely’).

The language of the six Choosing Wisely recommendations was modified on four factors (Table 1):

i) Providing less detail (‘what’ the recommendation is) vs. more detail (‘what’ the recommendation is, ‘who’ the recommendation is for, and ‘why’ the recommendation is important).
ii) Using unqualified (e.g. ‘don't…’) vs. qualified language (e.g. ‘don't routinely…’)
iii) Providing positive (‘do X’) vs. negative framing (‘don't do Y’); and
iv) Providing alternatives to low-value care vs. not providing alternatives.

**Table 1:** Language characteristics and how they are varied for the best-worst scaling survey.

Language characteristics	How it is varied	Examples
Specificity of the language	Specifying the ‘what’ only	***Don't request imaging for patients with non-specific low back pain and no indicators of a serious cause for low back pain.***
	Specifying the ‘what’, ‘who’ and ‘why’	***Physiotherapists should not request imaging for patients with non-specific low back pain and no indicators of a serious cause for low back pain as the findings are unlikely to positively guide management***
Qualification of the language	Using unqualified wording (‘don't’)	*Don't request imaging of the cervical spine in trauma patients, unless indicated by a validated decision rule.*
	Using qualified wording (‘don't routinely’)	***Consider avoiding imaging of the cervical spine in trauma patients, unless indicated by a validated decision rule.***
Positive or negative framing	Negative framing (“don't”)	*Don't request imaging for acute ankle trauma unless indicated by the Ottawa Ankle Rules.*
	Positive framing (“do”)	*Request imagining for acute ankle trauma when****indicated by the Ottawa Ankle Rules.***
Providing alternatives to low-value care	No alternative	*Avoid using electrotherapy modalities in the management of patients with low back pain.*
	Alternative mentioned	***Avoid using electrotherapy modalities in the management of patients with low back pain; instead, give****advice to stay active****and reassurance.***

To ensure readability and comprehensibility, we sought feedback from physical therapists on the re-worded Choosing Wisely recommendations. We conducted pilot testing with seven physical therapists to estimate how long it took participants to complete and assess comprehension. After the pilot testing, we decreased the number of questions from 15 in each block to 7 as the cognitive load of completing the survey was too high.

### Best-worst-scaling survey

**Figure 1:**
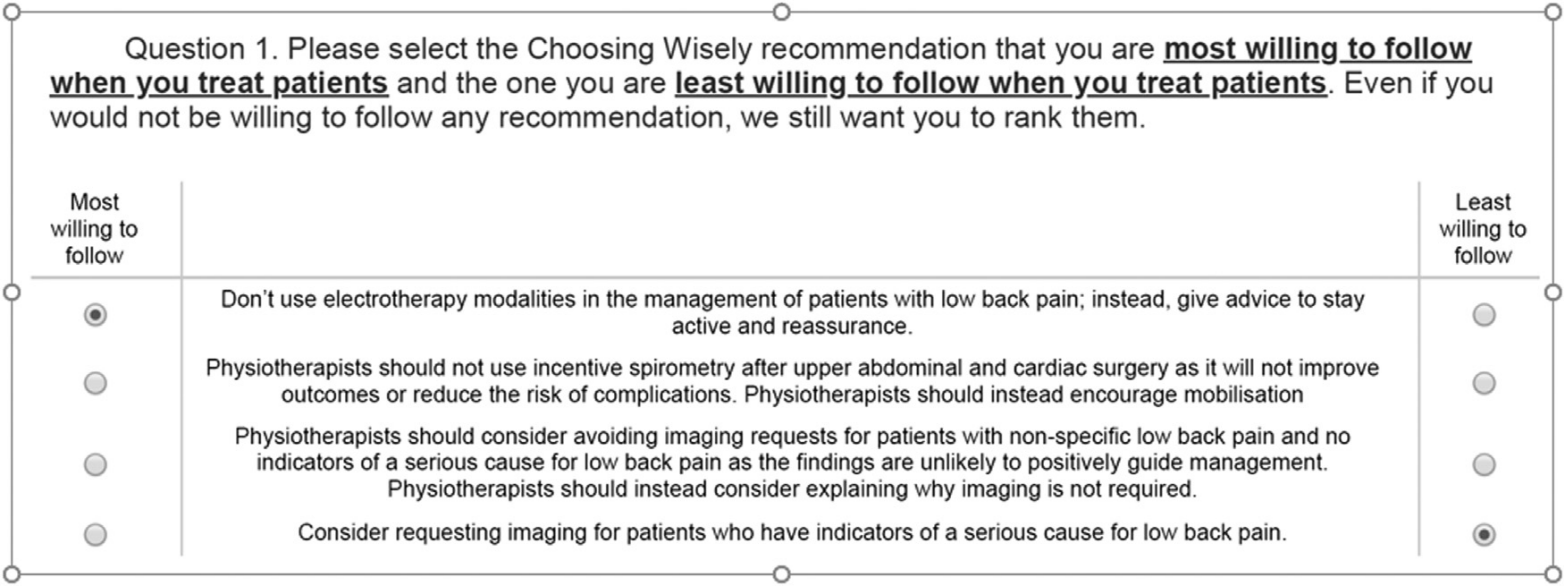
Example of a question from the best-worst scaling choice task.

Best-worst-scaling surveys are a type of ‘choice experiment’ that can be used to identify priorities/views and perspectives in healthcare.^17^ An object case best-worst-scaling survey was included consisting of 60 attributes (i.e., 60 recommendations; six original and 54 new recommendations) and used a balanced incomplete block design.^18^ We created 15 blocks each including seven choice tasks with four choice options (i.e., four recommendations). Each participant was randomised within the Qualtrics survey software to complete one block of seven choice tasks. Fig. 1 shows an example of one choice task. For each question, participants selected the recommendation they would be most willing and least willing to follow. There is no recognised approach for determining a minimum sample size for an object case best-worst scaling survey. Based on the experience of the researchers, a minimum sample size of 100 was considered appropriate to determine the main effects (i.e. relative importance of the attributes). However, larger sample sizes may be required to evaluate interaction effects.

### Data analysis

Survey responses were summarised using descriptive statistics (mean, median and standard deviations [SD], counts and percentages). We used a multinomial logistic (MNL) regression model to rank the 60 recommendations according to those participants who were most and least willing to follow them. Preference scores were based on the mean regression coefficients and 95% confidence intervals (CIs). For ease of interpretation, we calculated outcome level preference scores by normalizing the mean coefficients to a 0–10 scale, where 0 was the least and 10 the most preferred recommendation (‘normalised preference scores’) across all recommendations. Given the large number of recommendations, the marginal effects were calculated to assess the relative importance of different characteristics of the recommendations as well as the influence of sex, clinical area of interest, years of experience, familiarity with the recommendations, work setting, and involvement in research teaching or other professional activities on the preference scores. Marginal effects were calculated from the linear regression of preference scores with recommendation characteristics and subgroup analyses based on participant characteristics. A marginal effect describes the influence of the presence or absence of a characteristic on the preference score when all other variables are held at the average value. A positive value indicates that the characteristic increases preference scores while the opposite is the case for negative values. As all variables are on the same scale, the marginal effects can be directly compared thereby providing a basis for estimating the relative impact on preference across the 60 recommendations. We also described the ranking of the six original recommendations compared to the most preferred recommendation across the six topics of the original recommendations. Multinomial logit regression estimations were undertaken using NLOGIT V6 and linear regression and marginal effects using Stata Release 17.

## Results

### Participant characteristics

215 participants (48.5% of the 443 who opened the survey) completed the survey and could be included in the analysis. The mean age (SD) of the participants was 38.7 (10.6) years and 103 (47.9%) were female (Table 2). Most participants had ≥10 years of clinical experience (*n* = 123, 59.1%), worked in a private setting (*n* = 117, 55.2%) and worked as musculoskeletal physical therapists (*n* = 187, 88.2%). Half were at least slightly familiar with the Choosing Wisely recommendations (*n* = 107, 49.8%) and two-thirds (*n* = 139, 65%) were involved in research, teaching, or other professional activities.

**Table 2:** Characteristics of participants.

Characteristics	Mean (SD) or N (%)
Age *n* = 212	
Mean (SD) age (years)	**38.7 (10.6)**
20–29	54 (25.5%)
30–39	69 (32.5%)
40+	89 (42.0%)
Sex, *n* = 215	
Male	105 (48.8%)
Female	103 (47.9%)
Prefer not to say	4 (1.9%)
Not specified	3 (1.4%)
Country of practice *n* = 215	
Australia	64 (29.8%)
United States	37 (17.2%)
United Kingdom	30 (14.0%)
Canada	10 (4.7%)
Ireland	9 (4.1%)
Brazil	8 (3.7%)
Others	39 (18.1%)
Not specified	18 (8.4%)
Years of experience *n* = 212	
Mean (SD) years of experience	**14.2 (10.8)**
1–4 years	47 (22.2%)
5–9 years	41 (19.3%)
10+	124 (58.5%)
Clinical area of interest *n* = 212	
Musculoskeletal	131 (61.8%)
Cardiorespiratory	6 (2.8%)
Neurological	6 (2.8%)
Other^a^	69 (32.6%)
Setting *n* = 212	
Private Practice	107 (50.5%)
Public Hospital	66 (31.1%)
Private Hospital	10 (4.7%)
Aged Care	1 (0.5%)
Sports team	5 (2.4%)
Other	23 (10.9%)
Familiar with the APA's Choosing Wisely recommendation *n* = 215	
Extremely familiar	7 (3.3%)
Very familiar	22 (10.2%)
Moderately familiar	45 (20.9%)
Slightly familiar	33 (15.4%)
Not familiar at all	108 (50.2%)
Involvement in research, teaching or other professional activities *n* = 214	
Yes	139 (65.0%)
No	75 (35.0%)

*N*= total number of participants; n – number of participants who responded to the question; SD – Standard Deviation.

a ‘Other’ included: chronic pain, emergency medicine, frailty, gerontology, nutrition, hands, injury prevention, occupational health, orthomolecular medicine, orthopaedics, paediatrics, pain management, pelvic floor, pelvic health, primary care, rehabilitation, sports science, trauma, vestibular, women's health.

### Overall rank of recommendations

The top 10 and bottom 10 recommendations (based on preference scores) are presented in Table 3. A comparison between the original APA recommendations and the most preferred new recommendations (for each test and treatment) is shown in Table 4.

**Table 3:** Ranking of the recommendations based on preference scores (scaled from 0 to 10).





*The preference score coefficients were adjusted to a 0 to 10 scale.

^a^Text expressing different language characteristics is ***bolded and italicised***.

**Table 4:** Summary of the six original Choosing Wisely recommendations and the recommendations physical therapists are most willing to follow from the best-worst-scaling survey.

	Current Choosing Wisely Recommendations			Alternative Recommendations based on Preference Scores	
Recommendations type	Current APA recommendation	Language characteristics within the current recommendation	Rank among similar recommendations	Recommendations physical therapists are most willing to follow	Language characteristics within the most preferred recommendation
Imaging for low back pain	Don't request imaging for patients with non-specific low back pain and no indicators of a serious cause for low back pain.	Level of detail: Low Strength of language: Unqualified Framing: Negative Alternative: No	8th out of 12 recommendations	Physiotherapists should not request imaging for patients with non-specific low back pain and no indicators of a serious cause for low back pain as the findings are unlikely to positively guide management. Physiotherapists should instead explain why imaging is not required.	Level of detail: High Strength of language: Unqualified Framing: Negative Alternative: Yes
Electrotherapy for low back pain	Avoid using electrotherapy modalities in the management of patients with low back pain.	Level of detail: Low Strength of language: Qualified Framing: Negative Alternative: No	7th out of 8 recommendations	Physiotherapists should not use electrotherapy modalities in the management of patients with low back pain as they are not superior to placebo. Physiotherapists should instead give advice to stay active and reassurance	Level of detail: High Strength of language: Unqualified Framing: Negative Alternative: Yes
Imaging for acute ankle trauma	Don't request imaging for acute ankle trauma unless indicated by the Ottawa Ankle Rules.	Level of detail: Low Strength of language: Unqualified Framing: Negative Alternative: No	12th out of 12 recommendations	Physiotherapists should request imaging for acute ankle trauma when indicated by the Ottawa Ankle Rules as the findings could positively guide management	Level of detail: High Strength of language: Unqualified Framing: Positive Alternative: No
Imaging for cervical spine trauma	Don't request imaging of the cervical spine in trauma patients, unless indicated by a validated decision rule.	Level of detail: Low Strength of language: Unqualified Framing: Negative Alternative: No	8th out of 12 recommendations	Request imaging of the cervical spine in trauma patients if indicated by a validated decision rule.	Level of detail: Low Strength of language: Unqualified Framing: Positive Alternative: No
Manual therapy for adhesive capsulitis	Don't provide ongoing manual therapy for patients with adhesive capsulitis of the shoulder.	Level of detail: Low Strength of language: Unqualified Framing: Negative Alternative: No	7th out of 8 recommendations	Physiotherapists should consider avoiding ongoing manual therapy for patients with adhesive capsulitis of the shoulder as it is unlikely to improve recovery. Physiotherapists should instead consider providing reassurance and watchful waiting.	Level of detail: High Strength of language: Qualified Framing: Negative Alternative: Yes
Incentive spirometry after upper abdominal and cardiac surgery	Don't routinely use incentive spirometry after upper abdominal and cardiac surgery	Level of detail: Low Strength of language: Qualified Framing: Negative Alternative: No	7th out of 7 recommendations	Physiotherapists should not use incentive spirometry after upper abdominal and cardiac surgery as it will not improve outcomes or reduce the risk of complications. Physiotherapists should instead encourage mobilization	Level of detail: High Strength of language: Unqualified Framing: Negative Alternative: Yes

### Marginal effects of recommendations characteristics on preference scores

**Figure 2:**
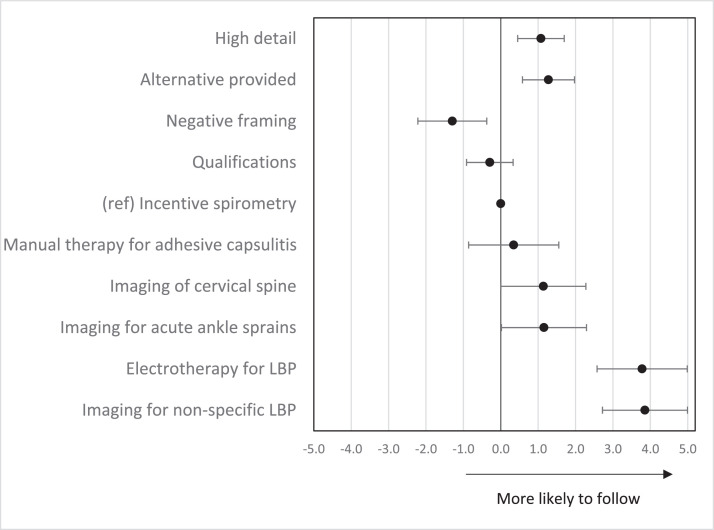
Marginal effects on preference scores (95% Confidence Interval) by recommendation type and characteristics. 95% Confidence Intervals that cross 0 suggest there is no effect.

Physical therapists were more willing to follow recommendations that provided alternatives (vs. no alternatives) to low-value care (1.3; 95% CI: 0.6, 2.0) and those with more detail (vs. less detail) (1.1; 95% CI: 0.5, 1.7), and less willing to follow recommendations that were negatively (vs. positively) framed (−1.3; 95% CI: −2.2, −0.4). The qualification of language did not influence physical therapists' willingness to follow recommendations (−0.3; 95% CI: −0.9, 0.3) (Fig. 2). Compared to the recommendation on incentive spirometry after upper abdominal and cardiac surgery, physical therapists were 40% more willing to follow recommendations to avoid imaging for low back pain (marginal effect 3.9; 95% CI: 2.7, 5.0) and electrotherapy for low back pain (3.8; 95% CI: 2.6, 5.0), and only 12% more willing to follow recommendations to avoid imaging for acute ankle trauma (1.2; 95% CI: 0.0, 2.3) and imaging of the cervical spine (1.1; 95% CI: 0.0, 2.3).

### Influence of physical therapists' characteristics on preference scores (sub-group analysis)

Physical therapists’ characteristics did not influence their willingness to follow recommendations that were negatively (vs. positively) framed or provided more (vs. less) detail. Willingness to follow recommendations with alternatives to low-value care was lower among physical therapists with less than 10 years of experience (0.7 vs. overall sample 1.3) and those who were not familiar with the recommendations (0.6 vs. overall sample 1.3). Willingness to follow qualified (‘avoid’, ‘don't routinely’) recommendations was lower among physical therapists working outside the private sector and those who had less than 10 years of experience, and higher among non-musculoskeletal physical therapists, with marginal effects on preference scores of −0.8, −0.5 and 0.6 (respectively) (Supplementary Material - Fig. S1) compared with the marginal effects on overall preference score for qualified recommendations (−0.29) (Supplementary Material - Table S1).

Willingness to follow recommendations to avoid imaging for non-specific low back pain (Type 1), electrotherapy for low back pain (Type 2) and manual therapy for adhesive capsulitis (Type 5) was lower among physical therapists working outside of musculoskeletal healthcare compared to the overall sample, with marginal effects on preference scores of 2.6 (95% CI: 1.2 to 4.0) vs 3.9 (95% CI: 2.7 to 5.0), 1.7 (95% CI: 0.3 to 3.2) vs. 3.8 (95% CI: 2.6 to 5.0) and −0.6 (95% CI: −2.1 to 0.8) vs. 0.3 (95% CI: −0.9 to 1.6), respectively (Supplementary Material - Figure S1) (Supplementary Material - Table S1).

## Discussion

### Summary of main findings

Overall, physical therapists were most willing to follow Choosing Wisely recommendations with more detail, and recommendations that provided alternatives to low-value care. While the qualification of the language used in recommendations did not affect physical therapists’ willingness to follow them, physical therapists were less willing to follow recommendations that were negatively framed. Physical therapists were most willing to follow recommendations that advised against imaging for non-specific low back pain and electrotherapy for low back pain. In the sub-group analysis, physical therapists working in the private sector were more willing to follow qualified recommendations compared to physical therapists working outside the private sector. Non-musculoskeletal physical therapists (vs. musculoskeletal physical therapists) were less willing to follow recommendations that advised against imaging for non-specific low back pain, electrotherapy for low back pain and manual therapy for adhesive capsulitis, and more willing to follow qualified (‘avoid’, ‘don't routinely’) recommendations.

### Strengths and limitations

To ensure we received a diverse range of opinions regarding the language of Choosing Wisely recommendations, we did not restrict participants based on their age, sex, clinical experience, or area of speciality, and we recruited physical therapists from all over the world (30 countries). The Best-Worst-Scaling design allowed us to identify which characteristics of language were likely to have the most influence on physical therapists’ willingness to follow Choosing Wisely recommendations. Best-Worst-Scaling surveys have been shown to have distinct advantages over traditional choice experiments (e.g., discrete choice experiments^19^) as they allow participants to select extremes (best and worst options), present a more clinically applicable choice task, and allow for greater insight into participants’ decision making.^20^

Our study has some limitations. Because demographics were assessed after the choice tasks, we do not have demographic data on the 228/443 participants (51.5%) who opened the link but did not complete the survey and thus, we cannot determine whether our sample is representative of the physical therapists who were initially willing to complete the survey. Another limitation is that we could only create 12 positively framed recommendations (out of 60) due to the wording of the initial recommendations. As a result, our findings may have underestimated or overestimated the benefit of positive framing.

### Meaning of the study

Our study highlights important aspects of language that could influence physical therapists’ willingness to follow Choosing Wisely recommendations and serve as a guide for writing future recommendations. Physical therapists were more likely to follow recommendations with more detail and recommendations that were positively framed, regardless of physical therapists’ characteristics or background (such as the clinical area of interest, years of experience, familiarity with the recommendations, and work setting). In contrast, all six of the APA's original recommendations were low on detail (i.e. only described the recommendation, and not ‘who’ it was for and ‘why’ it was important) and were negatively framed. These findings could explain why none of the APA's original recommendations made the top 10 most preferred recommendations.

Choosing Wisely, a global initiative with over 1300 recommendations, aims to make clinicians aware of avoiding low-value tests or treatments that do not benefit patients or sometimes even lead to harm.^21^ Our study showed that physical therapists were more willing to follow recommendations that were positively framed (vs. negatively framed) or provided alternatives (vs. no alternatives) to low-value care. The analysis showed that negative framing and providing alternatives to low-value care had a marginal effect of −1.3 and 1.3 on the preference scores (range 0 to 10), which implies that if everything were framed positively then this could increase physical therapists’ willingness to follow recommendations by 13% or if the recommendations provided alternatives to low-value care, then this would increase physical therapists’ willingness to follow recommendations by 13%. Developing Choosing Wisely lists involves a systematic process that considers the views and opinions of society members, associates, directors, specialists from the respective profession, and expert panels. Thus, it would be beneficial to discuss the findings with the Choosing Wisely team as more than 93% of these recommendations are negatively framed^22^ and only 4% provide alternatives to low-value care.^22^ When making future recommendations, care should be taken to ensure that the recommendations are positively framed or provide alternatives to low-value care as using negative framing or not providing alternatives to low-value care could be limiting the impact of the campaign.

Recommendations against imaging for non-specific low back pain and electrotherapy for low back pain were the most preferred recommendations. This finding is similar to the content analysis where feedback on a draft list of the APA Choosing Wisely recommendations was sought from 543 physical therapists.^14^ The study found most physical therapists agree that health professionals should avoid imaging for non-specific low back pain (75%) and electrotherapy for low back pain (52%).^14^ These interventions are well-recognised and accepted examples of low-value care as most guidelines for low back pain discourage both interventions.^23^ Many professional societies have targeted unnecessary imaging for non-specific low back pain in their Choosing Wisely lists, such as the Canadian Association of Emergency Physicians, Italian College of General Practice and Primary Care and the Royal College of Radiologists, United Kingdom.^22^ In the physical therapy community, there is also increasing recognition of the need to move away from providing passive modalities for low back pain and towards active care and self-management.^24^ This explains why some audits of physical therapy practice show that only a small percentage of physical therapists provide electrotherapy for low back pain.^3^

In the sub-group analyses, we found that musculoskeletal physical therapists were more willing to follow recommendations against imaging for non-specific low back pain and electrotherapy for low back pain when compared with non-musculoskeletal physical therapists. This could be because musculoskeletal physical therapists were more familiar with recommendations that advised against imaging for non-specific low back pain, electrotherapy for low back pain and manual therapy for adhesive capsulitis, as these are some of the major examples of low-value care in this area of practice.

### Comparison with previous research

Physical therapists were more willing to follow recommendations that were more detailed (i.e. specified ‘what’ the recommendation was, ‘who’ it was for and ‘why’ it was important) vs. less details (i.e. only specified ‘what’). This aligns with the findings of the content analysis where physical therapists provided feedback on a draft list of the APA's Choosing Wisely recommendations. In this study physical therapists suggested that recommendations need more detail to increase implementation.^14^ Previous studies investigating the effects of language on guideline implementation and clinician/patient behavior also show similar results.^10^, ^11^, ^12^ For example, a randomised controlled trial of 84 mental health service patients investigated the effect of improving the readability of the National Institute of Clinical Excellence (NICE) guidelines for the management of schizophrenia on guideline implementation.^25^ Making simple amendments to the guidelines (e.g. made easier to read, understand and act upon) improved patient attitudes towards the guideline and intention to implement the recommendations.^25^ Similarly, a vignette-based trial found specific (vs. non-specific) guidelines for the management of low back pain increased appropriate ordering of electrodiagnostic tests and reduced inappropriate ordering among general internists, neurologists, and physical medicine specialists.^11^ A study examining the influence of guidelines attributes on clinical decision-making^10^ found that 67% of general practitioners (*n* = 41/61) follow recommendations that are clear, detailed and specific compared to only 36% who follow recommendations that are unclear and non-specific.^10^ Similarly, a systematic meta-review (12 systematic reviews exploring factors influencing the implementation of clinical guidelines) showed that guidelines that were clear and easy to understand were more likely to be implemented by health professionals.^26^

### Implications for future research

There has been a shift in clinical practice guideline recommendations for musculoskeletal conditions over the last few decades away from recommendations for medicines and surgery and instead toward physical and psychological management. This shift has and will likely continue to result in more people with musculoskeletal conditions seeking treatment from physical therapists. It is thus important to consider strategies that can guide physical therapists away from providing low-value care. Our study highlights that refining the original Choosing Wisely recommendations - by providing more detail, using positive framing where possible, and providing alternatives to low-value care – is an important step towards increasing adoption of these recommendations among physical therapists, and more broadly future studies could explore how this simple, low-cost strategy could support the adoption of recommendations.

## Conclusion

Recommendations which were positively framed, included more detail and provided alternatives to low-value care were more likely to be followed by physical therapists. These findings demonstrate the ability of language to influence willingness and support the need to modify the language of future and existing Choosing Wisely recommendations. Optimizing the language of Choosing Wisely recommendations could increase their implementation among physical therapists and health professionals more broadly and help reduce low-value care provided to patients.

## Declaration of Competing Interest

None declared.

## Appendix Supplementary materials

Image, application 1

## Footnotes

Supplementary material associated with this article can be found in the online version at doi:10.1016/j.bjpt.2023.100534.
