# Supplementary material for: Can language enhance physical therapists’ willingness to follow Choosing Wisely recommendations? A best-worst scaling study
Source: Braz J Phys Ther. 2023 Aug 14;27(4):100534. doi: 10.1016/j.bjpt.2023.100534 (PMC10462803; doi:10.1016/j.bjpt.2023.100534)
Supplement: Supplementary file 1 [file mmc1.pdf]

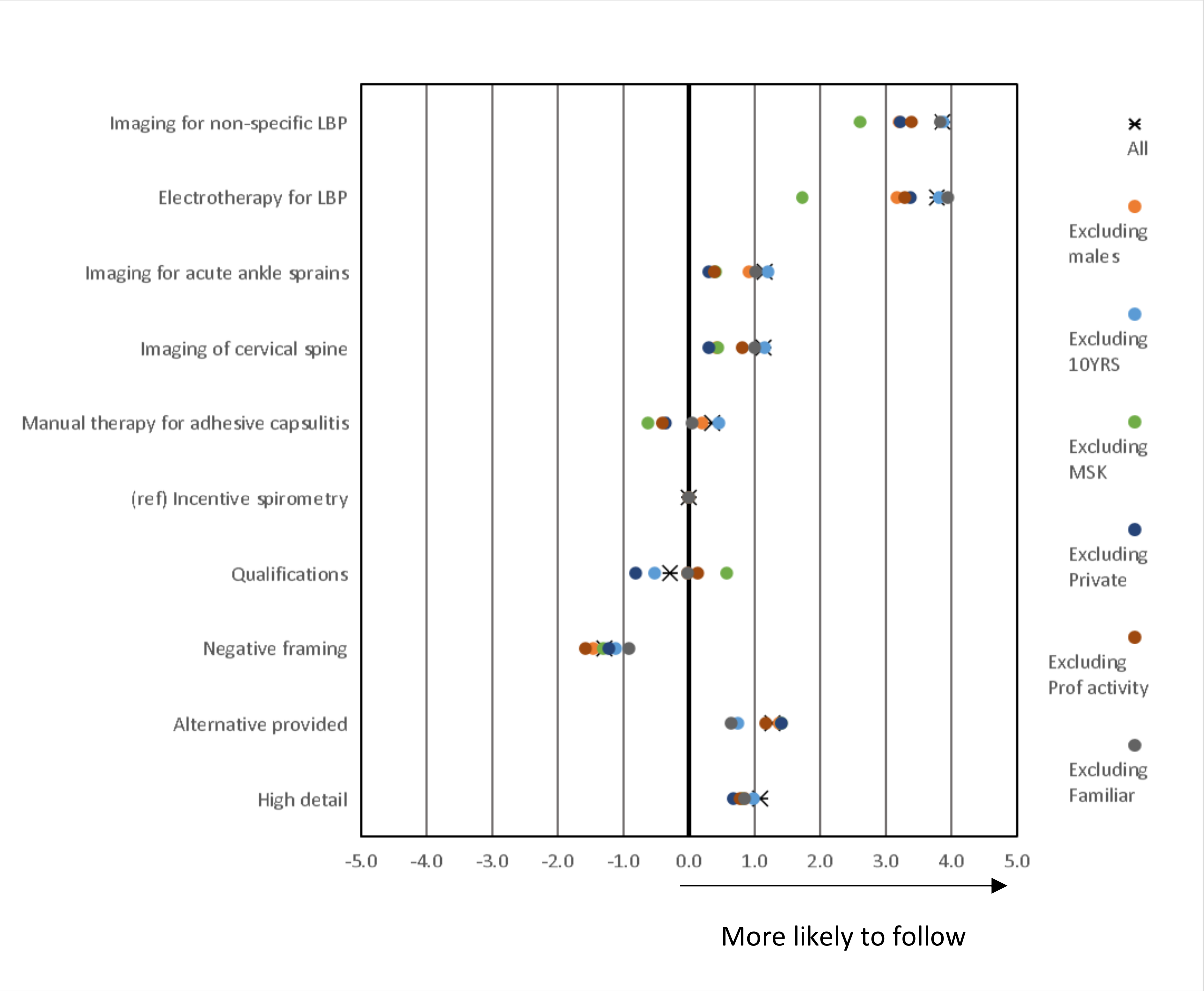

Supplementary Material - Figure S1: Effect of respondents' characteristics on the willingness to follow the recommendations

*10YRS: those with ≥10 years of experience; MSK: musculoskeletal physiotherapists; Private: those who worked in private hospitals or clinics; Prof act: those involved in research teaching or other professional activities; Familiar: those who were slightly/moderately/very/extremely familiar with the recommendations.

Supplementary Material - File S1: Survey questions

1. PARTICIPANT CONSENT FORM

Exploring the Language of Choosing Wisely Recommendations.

PARTICIPANT CONSENT STATEMENT

In giving my consent I state that:

• I have read the Participant Information Statement and have been given the opportunity to discuss the study and my involvement in it with the researcher/s.
• The procedures required and time involved (including any inconvenience, risk, discomfort or side effect, and their implications) have been explained to me, and my questions about the project have been answered to my satisfaction.
• I understand that participation is voluntary. I am under no obligation to consent.
• I understand that I can withdraw from the study at any time, without providing a reason and without suffering any penalty. This will not affect my relationship with the researcher/s or university.
• I understand that my involvement is strictly confidential and no information about me will be used in any way that reveals my identity.
• I understand that data from this study may be used again for future research purposes, but that all data is strictly confidential and no information about me will be used in any way that reveals my identity.

- ☐ Yes, I would be happy to go on and complete the survey
- ☐ No, I would prefer not to complete the survey

2. I consent to being contacted about future studies.
   - ☐ Yes
   - ☐ No
3. I would like to receive overall results of this study.
   - ☐ Yes
   - ☐ No
4. Please provide your contact details if you indicated that you would like to be contacted about future studies or receive overall results of this study. Your details will be stored securely and kept separate from your questionnaire responses, which will be stored as anonymous.
5. First Initial, last initial and year of birth (E.g., JR 1982)

____

6. Email address or mailing address

____

First some clinical questions…

7. How would you rate your knowledge in the following areas?

	Extremely knowledgeable	Very knowledgeable	Moderately knowledgeable	Slightly knowledgeable	Not knowledgeable at all
Imaging guidelines for low back pain					
Imaging guidelines following cervical spine trauma					
Imaging guidelines following ankle trauma					
Indications for incentive spirometry					
Evidence on the effectiveness of electrotherapy for low back pain					
Evidence on the effectiveness of manual therapy for adhesive capsulitis (frozen shoulder)					

8. How would you rate your clinical expertise in the following areas?

	Excellent	Good	Average	Poor	Terrible
Assessment and management of low back pain					
Assessment and management following cervical spine trauma					
Assessment and management following ankle trauma					
Cardiorespiratory physiotherapy					

Assessment and management of adhesive capsulitis (frozen shoulder)					

9. Please rate your agreement with the following Choosing Wisely recommendations for physiotherapists.

	Strongly agree	Somewhat agree	Neither agree nor disagree	Somewhat disagree	Strongly disagree
**Don’t request imaging for patients with non-specific low back pain and no indicators of a serious cause for low back pain**					
D**on’t request imaging of the cervical spine in trauma patients, unless indicated by a validated decision rule**					
**Don’t request imaging for acute ankle trauma unless indicated by the Ottawa Ankle Rules. (localized bone tenderness or inability to weight-bear as defined in the Rules)**					
**Don't routinely use incentive spirometry after upper abdominal and cardiac surgery**					
**Avoid using electrotherapy modalities in the management of patients with low back pain**					
**Don’t provide ongoing manual therapy for patients with adhesive capsulitis of the shoulder**					

10. How familiar were you with the Australia Physiotherapy Association’s Choosing Wisely recommendations prior to being invited to complete this survey?
    - ☐ Extremely familiar
    - ☐ Very familiar
    - ☐ Moderately familiar
    - ☐ Slightly familiar
    - ☐ Not familiar at all

<u>Best-Worst-Scaling Survey explanation</u>

In the next 7 questions you will be shown a list of Choosing Wisely recommendations. There will be a mix of the original and modified recommendations.

Please read each recommendation carefully. Some recommendations might appear the same but they are worded slightly differently.

From each list we would like you to **<u>select the Choosing Wisely recommendation that you are most willing to follow</u>** when you treat patients **<u>and the one you are least willing to follow</u>** when you treat patients. Even if you would not be willing to follow any recommendation, we still want you to rank them.

If you are completing this survey on a mobile, using landscape view might make it easier to answer the following questions.

An example of a completed question is shown below. Please proceed to the actual survey on the next screen when you are ready.

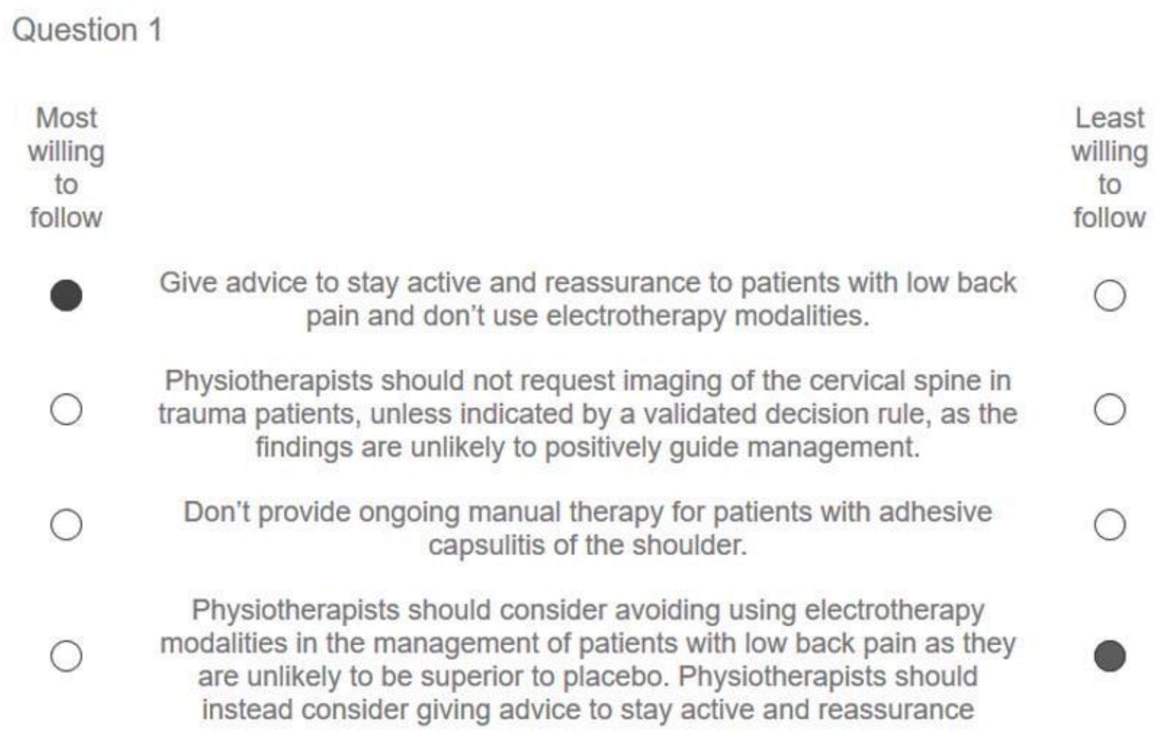
Question 1

Most willing to follow		Least willing to follow
●	Give advice to stay active and reassurance to patients with low back pain and don't use electrotherapy modalities.	○
○	Physiotherapists should not request imaging of the cervical spine in trauma patients, unless indicated by a validated decision rule, as the findings are unlikely to positively guide management.	○
○	Don't provide ongoing manual therapy for patients with adhesive capsulitis of the shoulder.	○
○	Physiotherapists should consider avoiding using electrotherapy modalities in the management of patients with low back pain as they are unlikely to be superior to placebo. Physiotherapists should instead consider giving advice to stay active and reassurance	●

Best-Worst-Scaling Survey

[example set of 7 questions]

Question 1

Most willing to follow		Least willing to follow
◯	Give advice to stay active and reassurance to patients with low back pain and don't use electrotherapy modalities.	◯
◯	Physiotherapists should not request imaging of the cervical spine in trauma patients, unless indicated by a validated decision rule, as the findings are unlikely to positively guide management.	◯
◯	Don't provide ongoing manual therapy for patients with adhesive capsulitis of the shoulder.	◯
◯	Physiotherapists should consider avoiding using electrotherapy modalities in the management of patients with low back pain as they are unlikely to be superior to placebo. Physiotherapists should instead consider giving advice to stay active and reassurance	◯

Question 2

Most willing to follow		Least willing to follow
◯	Request imaging of the cervical spine in trauma patients if indicated by a validated decision rule.	◯
◯	Physiotherapists should not provide ongoing manual therapy for patients with adhesive capsulitis of the shoulder as there is no evidence it improves recovery. Physiotherapists should instead provide reassurance and watchful waiting.	◯
◯	When the Ottawa Ankle Rules are negative, explain why imaging is not required and don't request imaging.	◯
◯	Don't request imaging for acute ankle trauma unless indicated by the Ottawa Ankle Rules.	◯

Question 3

Most willing to follow		Least willing to follow
◯	Physiotherapists must not request imaging for acute ankle trauma unless indicated by the Ottawa Ankle Rules as the findings are unlikely to positively guide management. Physiotherapists must instead explain why imaging is not required.	◯
◯	Don’t provide ongoing manual therapy for patients with adhesive capsulitis of the shoulder.	◯
◯	Physiotherapists should consider avoiding ongoing manual therapy for patients with adhesive capsulitis of the shoulder as it is unlikely to improve recovery. Physiotherapists should instead consider providing reassurance and watchful waiting.	◯
◯	Physiotherapists should consider avoiding imaging of the cervical spine in trauma patients, unless indicated by a validated decision rule, as the findings are unlikely to positively guide management	◯

Question 4

Most willing to follow		Least willing to follow
◯	Physiotherapists should request imaging for patients who have indicators of a serious cause for low back pain as the findings could positively guide management	◯
◯	Consider avoiding imaging requests for patients with non-specific low back pain and no indicators of a serious cause for low back pain	◯
◯	Physiotherapists should consider requesting imaging for patients who have indicators of a serious cause for low back pain as the findings could positively guide management.	◯
◯	When the Ottawa Ankle Rules are negative, consider explaining why imaging is not required and consider avoiding requesting imaging as the findings are unlikely to positively guide management	◯

Question 5

Most willing to follow		Least willing to follow
◯	When the Ottawa Ankle Rules are negative, physiotherapists must explain why imaging is not required and not request imaging as the findings are unlikely to positively guide management	◯
◯	Physiotherapists should encourage mobilisation after upper abdominal and cardiac surgery and not use incentive spirometry as it will not improve outcomes or reduce the risk of complications	◯
◯	Physiotherapists should consider avoiding imaging requests for acute ankle trauma unless indicated by the Ottawa Ankle Rules as the findings are unlikely to positively guide management. Physiotherapists should instead consider explaining why imaging is not required.	◯
◯	Physiotherapists must not use electrotherapy modalities in the management of patients with low back pain as they are not superior to placebo	◯

Question 6

Most willing to follow		Least willing to follow
◯	Don't use incentive spirometry after upper abdominal and cardiac surgery	◯
◯	Consider explaining why imaging is not required to patients with non-specific low back pain and no indicator of a serious cause for low back pain; and consider not requesting imaging.	◯
◯	Physiotherapists must not request imaging for patients with non-specific low back pain and no indicators of a serious cause for low back pain as the findings are unlikely to positively guide management. Physiotherapists must instead explain why imaging is not required.	◯
◯	When a validated decision rule for imaging patients with cervical spine trauma is negative, physiotherapists should consider explaining why imaging is not required and consider avoiding requesting imaging as the findings are unlikely to positively guide management.	◯

Question 7

Most willing to follow		Least willing to follow
○	Physiotherapists must provide reassurance and watchful waiting to patients with adhesive capsulitis of the shoulder and must not provide ongoing manual therapy as there is no evidence it improves recovery	○
○	When the Ottawa Ankle Rules are negative, physiotherapists must explain why imaging is not required and not request imaging as the findings are unlikely to positively guide management	○
○	Request imaging for patients who have indicators of a serious cause for low back pain	○
○	Physiotherapists should consider avoiding imaging of the cervical spine in trauma patients, unless indicated by a validated decision rule, as the findings are unlikely to positively guide management. Physiotherapists should instead consider explaining why imaging is not required.	○

<u>Finally some quick questions about you...</u>

11. Please indicate your gender:
    - ☐ Female
    - ☐ Male
    - ☐ Prefer not to say
12. Please indicate your age:

    ____

13. Please indicate your country of birth:
    - ☐ Australia
    - ☐ Other (please specify) ___________________
14. What qualifications do you have relevant to physiotherapy? (select as many as apply)
    - ☐ Bachelors
    - ☐ Masters
    - ☐ PhD
    - ☐ Titling
    - ☐ Specialisation
    - ☐ Other (please specify) ______________________________
15. In which country did you receive your qualification(s)?
    - ☐ Australia
    - ☐ Other (please specify) ___________________
16. How many years have you been practising for?

    ____

17. In which country do you practice:
    - ☐ Australia
    - ☐ Other (please specify) ___________________
18. What do you consider your clinical area(s) of interest? (select as many as apply)
    - ☐ Musculoskeletal
    - ☐ Cardiorespiratory
    - ☐ Neurological
    - ☐ Other (please specify) _____________________________
19. Which clinical setting have you spent the most time practising in?
    - ☐ Private practice
    - ☐ Public hospital
    - ☐ Private hospital
    - ☐ Aged care
    - ☐ Sports teams
    - ☐ Other (please specify) _____________________________
20. Which clinical setting are you currently practicing in? (select as many as apply)
    - ☐ Private practice
    - ☐ Public hospital
    - ☐ Private hospital
    - ☐ Aged care
    - ☐ Sports teams
    - ☐ Other (please specify) _____________________________
21. Are you currently involved in any of the following professional activities? (select as many as apply)
    - ☐ Research
    - ☐ Teaching physiotherapy students
    - ☐ Teaching continuing education courses
    - ☐ Other (please specify) _____________________________
    - ☐ None

Supplementary Material - Table S1: Marginal effects on preference scores

	Marginal effect	Std. Err.	P	95% LCL	95% UCL
**All**					
Type 1	3.86	0.57	0.00	2.72	4.99
Type 2	3.78	0.60	0.00	2.58	4.99
Type 3	1.16	0.57	0.05	0.02	2.29
Type 4	1.14	0.57	0.05	0.00	2.28
Type 5	0.35	0.60	0.57	-0.86	1.55
Type 6	0.00				
Qualifications	-0.29	0.31	0.35	-0.92	0.33
Negative framing	-1.30	0.46	0.01	-2.22	-0.37
Alternative provided	1.28	0.35	0.00	0.58	1.97
High detail	1.07	0.31	0.00	0.45	1.70
**Excluding males**					
Type 1	3.21	0.65	0.00	1.91	4.51
Type 2	3.16	0.69	0.00	1.78	4.54
Type 3	0.91	0.65	0.17	-0.39	2.21
Type 4	0.42	0.65	0.52	-0.89	1.72
Type 5	0.21	0.69	0.77	-1.17	1.59
Type 6	0.00				
Qualifications	-0.02	0.35	0.95	-0.73	0.69
Negative framing	-1.46	0.52	0.01	-2.51	-0.41
Alternative provided	1.37	0.40	0.00	0.57	2.16
High detail	0.84	0.35	0.02	0.13	1.55
**Excluding YR3**					
Type 1	3.88	0.62	0.00	2.64	5.12
Type 2	3.81	0.65	0.00	2.50	5.12
Type 3	1.20	0.62	0.06	-0.04	2.44
Type 4	1.16	0.62	0.07	-0.08	2.39
Type 5	0.46	0.65	0.49	-0.86	1.77
Type 6	0.00				
Qualifications	-0.52	0.34	0.13	-1.20	0.16
Negative framing	-1.11	0.50	0.03	-2.12	-0.11
Alternative provided	0.74	0.38	0.06	-0.02	1.50
High detail	0.98	0.34	0.01	0.30	1.66
**Excluding MSK**					
Type 1	2.60	0.68	0.00	1.25	3.96
Type 2	1.72	0.72	0.02	0.28	3.16
Type 3	0.41	0.68	0.55	-0.95	1.77
Type 4	0.45	0.68	0.51	-0.91	1.80
Type 5	-0.62	0.72	0.39	-2.06	0.82

	Marginal effect	Std. Err.	P	95% LCL	95% UCL
Type 6	0.00				
Qualifications	0.57	0.37	0.13	-0.17	1.32
Negative framing	-1.31	0.55	0.02	-2.41	-0.21
Alternative provided	1.41	0.41	0.00	0.58	2.24
High detail	0.81	0.37	0.03	0.07	1.55
**Excluding Private**					
Type 1	3.22	0.84	0.00	1.53	4.91
Type 2	3.36	0.89	0.00	1.57	5.16
Type 3	0.30	0.84	0.73	-1.39	1.98
Type 4	0.30	0.84	0.73	-1.39	1.99
Type 5	-0.36	0.89	0.68	-2.16	1.43
Type 6	0.00				
Qualifications	-0.82	0.46	0.08	-1.75	0.10
Negative framing	-1.23	0.68	0.08	-2.59	0.14
Alternative provided	1.40	0.51	0.01	0.36	2.43
High detail	0.68	0.46	0.14	-0.24	1.61
**Excluding PRF**					
Type 1	3.38	0.63	0.00	2.12	4.65
Type 2	3.29	0.67	0.00	1.95	4.63
Type 3	0.39	0.63	0.54	-0.88	1.65
Type 4	0.80	0.63	0.21	-0.46	2.07
Type 5	-0.42	0.67	0.54	-1.75	0.92
Type 6	0.00				
Qualifications	0.14	0.34	0.68	-0.55	0.83
Negative framing	-1.58	0.51	0.00	-2.61	-0.56
Alternative provided	1.16	0.39	0.00	0.39	1.93
High detail	0.77	0.34	0.03	0.08	1.46
**Excluding FMLR**					
Type 1	3.83	0.68	0.00	2.48	5.19
Type 2	3.95	0.72	0.00	2.51	5.39
Type 3	1.02	0.68	0.14	-0.34	2.37
Type 4	1.00	0.68	0.14	-0.35	2.36
Type 5	0.04	0.72	0.95	-1.40	1.48
Type 6	0.00				
Qualifications	-0.01	0.37	0.97	-0.76	0.73
Negative framing	-0.91	0.55	0.10	-2.01	0.19
Alternative provided	0.64	0.41	0.13	-0.19	1.47
High detail	0.85	0.37	0.03	0.11	1.59
